# Testing the feasibility of an intermittent low‐energy diet in women with gestational diabetes

**DOI:** 10.1111/dme.70258

**Published:** 2026-03-20

**Authors:** Elizabeth Dapre, Emma Barrett, Brian McMillan, Andrea Pilkington, Fahmy Hanna, Avni Vyas, Cheryl Lombardelli, Womba Mubita, Jessy Athirampuzha, Liveen Dosanjh, Michelle Harvie, Basil G. Issa

**Affiliations:** ^1^ Centre for Primary Care and Health Services Research The University of Manchester Manchester UK; ^2^ Centre for Biostatistics The University of Manchester Manchester UK; ^3^ Obstetrics and Gynaecology Manchester University NHS Foundation Trust Manchester UK; ^4^ Diabetes and Endocrinology University Hospitals of North Midlands NHS Trust Stoke‐on‐Trent UK; ^5^ Faculty of Health and Education, Department of Health Professions Manchester Metropolitan University Manchester UK; ^6^ Department of Nutrition and Dietetics Manchester University NHS Foundation Trust, Manchester Academic Health Science Centre Manchester UK; ^7^ Diabetes, Endocrinology and Metabolic Services Manchester University NHS Foundation Trust Manchester UK; ^8^ School of Medical Sciences The University of Manchester Manchester UK; ^9^ Division of Cancer Sciences The University of Manchester Manchester UK

**Keywords:** exercise, feasibility, gestational diabetes, healthy diet, intermittent low energy diet, pregnancy, safety

## Abstract

**Aims:**

The study aimed to assess feasibility, acceptability and safety of an ILED in a randomised controlled trial of ILED vs. best NHS care healthy diet and exercise advice in women with Gestational Diabetes Mellitus (GDM) with body mass index ≥27.5 kg/m^2^ (≥25 kg/m^2^ in high‐risk minority ethnic groups) in the last trimester of pregnancy.

**Methods:**

Participants were randomised (1:1) to receive an ILED (1000 kcal on two non‐consecutive days of the week with 5 days/week of best NHS care healthy diet and exercise advice) or best NHS care healthy diet and exercise advice 7 days/week from recruitment (24–30 weeks gestation) until delivery (BNC). Primary outcomes were uptake, retention and adherence to the interventions. Maternal and neonatal safety outcomes were explored, and secondary outcomes assessed fidelity of the delivery of the intervention and completeness of study data across all trial participants.

**Results:**

Participants were randomised to ILED (*n* = 14) or BNC (*n* = 12) and 73% (*n* = 19; ILED *n* = 10, BNC *n* = 9) received their allocated intervention. There was low study retention (58%; ILED *n* = 7; BNC *n* = 8) and low adherence to the ILED median (IQR) 37% (8%–50%) out of prescribed days; however, the study achieved a feasible uptake rate (31%; *n* = 26). There were no adverse events specifically related to the ILED.

**Conclusion:**

Our findings suggest that ILED may not be feasible in the third trimester in people with GDM. Future research should explore alternative interventions and interventions earlier in pregnancy for the prevention and management of GDM.

**Trial registration number**: NCT05344066

**What's new?**

**What is already known?**

There is no strong evidence for a particular dietary regimen for glycaemic control in gestational diabetes (GDM). Intermittent low‐energy diets (ILED) are safe in people with type 2 diabetes and demonstrate comparable or greater reductions in weight, insulin resistance and hyperglycaemia than daily energy restriction.

**What is the key question?**

Is an ILED feasible, safe and acceptable in GDM?

**What this study found?**

There were no safety concerns with ILED. Adherence was poor with high attrition rates suggesting that ILED is not a feasible dietary intervention for GDM.

**What are the implications?**

Dietary adherence is a challenge during GDM. Future research should consider dietary interventions earlier in pregnancy.

## 1 INTRODUCTION

Gestational diabetes mellitus (GDM) is glucose intolerance which occurs during pregnancy, affecting around 5% of UK pregnancies.^1^ The incidence is rising, in part due to increasing rates of obesity and maternal age, and it is associated with maternal and neonatal complications including macrosomia, shoulder dystocia, caesarean sections, stillbirth and an increased lifetime risk of diabetes and cardiometabolic syndrome in mother and offspring.^1^, ^2^

First‐line therapy for GDM is diet and lifestyle modification. The National Institute for Health and Care Excellence (NICE) recommend personalised dietitic advice to follow a healthy diet, including increased fruit and vegetables, low‐glycaemic index foods, reduced refined sugars, regular mealtimes and regular exercise, with levels of dietetic support varying between units in the United Kingdom.^3^ This approach fails to achieve glycaemic targets in around 40% of women who progress to pharmacotherapy. A recent UK study found 59% of patients required metformin and 41% required insulin.^4^

There is no convincing evidence that a particular diet (e.g. low glycaemic index, low carbohydrate or modest energy‐restriction) improves outcomes in GDM.^5^, ^6^, ^7^ Some have shown promise in reducing gestational weight gain but have a limited impact on glycaemic control.^6^, ^7^ The optimum dietary regimen must promote adequate nutrition, appropriate weight gain, normoglycaemia and be acceptable to women.^8^ A possible dietary approach is an intermittent low‐energy diet (ILED) which includes several days of a low‐energy diet (650–1000 kcal) with normal eating on other days. These are considered safe in people with type 2 diabetes (T2DM)^9^ and are associated with comparable or greater reductions in weight, insulin resistance and hyperglycaemia than daily energy restriction in non‐pregnant populations.^10^, ^11^ To our knowledge, they have not been studied in a pregnant population.

This study aims to determine whether an ILED is feasible, acceptable and likely to be safe in the management of GDM compared to best NHS care (BNC) in a cohort of women with overweight or obesity.

## 2 METHODS

### 2.1 Study design and participants

This mixed methods feasibility randomised controlled trial was conducted at the maternity unit Manchester University NHS Foundation Trust (MFT). Study participants were recruited between November 2022 and December 2023 through routine antenatal clinics at 24–30 weeks gestation following a positive diagnosis of GDM. Participants were screened for eligibility by two midwives in the unit using standard antenatal booking weights from calibrated NHS scales, and included those with body mass index (BMI) ≥27.5 kg/m^2^, or ≥25 kg/m^2^ in high‐risk minority ethnic groups (i.e. South Asian, Black African and African Caribbean), singleton pregnancy, scheduled for first‐line diet and exercise management and not receiving anti‐diabetes medication (Table 1). The study was granted ethical approval by Cambridge East Ethics Committee (ref. 22/EE/0119) and the protocol is published in British Medical Journal Open.^12^

**TABLE 1:** Inclusion and exclusion criteria.

**Inclusion criteria**
Pregnant women ≥18 years old with singleton pregnancy Body mass index ≥27.5 kg/m^2^, or ≥25 kg/m^2^ in high‐risk minority ethnic groups (i.e. South Asian, Black African and African Caribbean) and <50 kg/m^2^ at booking appointment Newly diagnosed gestational diabetes mellitus (GDM) according to local diagnostic criteria (fasting glucose ≥5.3 mmol/L and/or 2‐h postprandial glucose ≥8.5 mmol/L with 75 g oral glucose tolerance test) Scheduled to receive first‐line NHS diet and exercise advice and not currently using anti‐diabetic medication 24–30 weeks pregnant
**Exclusion criteria**
Pre‐existing diabetes Fasting glucose ≥7.0 mmol/L or ≥11.0 mmol/L at OGTT requiring immediate medical intervention Significant comorbid disease (e.g. chronic kidney disease, significant cardiac disease, or severe psychological problems including disordered eating) Current participation in another GDM or dietary trial Inability to provide informed consent and/or adhere to safety protocols Previous bariatric surgery or weight loss medications Concurrent medications with potential to affect results (e.g. high dose steroids, immunosuppressants) Previous intrauterine growth restriction Weight loss >5% from antenatal booking to screening appointment

This paper reports feasibility outcomes (trial uptake, retention, ILED adherence and safety). The qualitative sub‐study of nine participants' and five healthcare professionals' views on feasibility and acceptability is under review elsewhere.

### 2.2 Study procedures

#### 2.2.1 Randomisation

Participants who gave informed consent were randomised (1:1) to ILED or BNC 7 days/week until delivery using Sealed Envelope software (Solar House, London, UK) by an independent researcher. Randomisation was stratified by age (18–35 and >35 years) and BMI (27.5–34.99 kg/m^2^ and >35 kg/m^2^, >25–32.49 kg/m^2^ and >32.5 kg/m^2^, respectively, for White and high‐risk minority ethnic groups). Participants and clinicians were unmasked to the intervention.

#### 2.2.2 Interventions

ILED and BNC diet composition are summarised in Table 2. Participants were asked to follow their allocated diet during pregnancy to optimise glycaemic control and limit weight gain. Both groups received a standard healthy diet advice booklet at diagnosis in line with national recommendations.^3^ Within one week of joining the study (gestational week 25–31) both groups received personalised diet and physical activity advice from their allocated study dietitian using motivational interviewing techniques.^13^ Dietetic support was matched between the groups and participants were offered scheduled fortnightly dietitian contact via face‐to‐face, telephone or video call between study recruitment and delivery (Appendix 1). Participants could request additional support where necessary and advice was tailored to different ethnic and dietary groups. Partcipants were encouraged to continue their allocated diet in the 12‐week postpartum but did not receive postpartum dietetic support. Dietetic support for both groups in the study is likely to be greater than that routinely offered in NHS clinics, but was used in the study to maximise adherence to the interventions.

**TABLE 2:** Composition of the diet and physical activity advice for the intermittent low‐energy diet (ILED) and Best NHS Care (BNC) groups.

ILED (2 × non‐consecutive 1000 kcal days)	ILED (5 BNC days/week)	BNC (7 days/week)
100 g low‐GI carbohydrate (4 portions carbohydrate foods)	Low‐GI foods, reduction in free sugars	Low ‐GI foods, reduction in free sugars
5 portions of vegetables, 2 portions of fruit	Increased fruit/vegetable intake	Increased fruit/vegetable intake
70 g protein (210 g lean protein foods)	≥70 g protein	≥70 g protein
3 portions dairy/dairy alternative		
~22 g fibre	≥28 g fibre	≥28 g fibre
7 g fat	Predominantly monounsaturated/polyunsaturated fats	Predominantly monounsaturated/polyunsaturated fats
Physical activity (~150 min moderate intensity physical activity/week; walking for 30 min after any meal wherever possible)	Physical activity (~150 min moderate intensity physical activity/week; walking for 30 min after any meal wherever possible)	Physical activity (~150 min moderate intensity physical activity/week; walking for 30 min after any meal wherever possible)

Abbreviation: GI, glycaemic index.

All participants received routine antenatal care and support from the study endocrinologist and specialist diabetes midwives in line with NICE guidelines, including routine growth scans, risk assessments and labour and delivery plans.^3^ Blood sugar was reviewed by the study endocrinologist two‐four weekly, and decisions regarding pharmacotherapy followed the study medication protocol (Table 3). Participant weight and blood pressure were measured four weekly (Appendix 1).

**TABLE 3:** Medical management protocol.

**Hypoglycaemia (blood glucose < 4 mmol/L)** Participants are advised to take 15–20 g of rapid acting carbohydrate which is anticipated to raise blood glucose by 3 mmol/L. Examples of rapid acting carbohydrate include 170–225 mL Lucozade Original (not Lucozade Sport), a small carton of fruit juice, 5–6 glucose tablets, 4/5 jelly babies or a small tin of coca cola (150–200 mL). Participants will be advised to repeat the treatment every 15 min until blood glucose is ≥4 mmol/L. If it is <1 h before the next meal participants are advised to try to avoid additional slow acting carbohydrate. If it is 1–2 h before the next meal participants are advised to take 10 g additional slow acting carbohydrate. Is it is >2 h before the next meal participants are advised to take 15–20 g additional slow acting carbohydrate.
**Ketonaemia** If ketone levels ≥1.0 mmol/L on a fasting sample: Drink 1 L fluids and repeat ketone levels at 4 h. If ketone level has improved (<1.0 mmol/L), no further action required. If ketone level has increased or remains the same, repeat ketone level after 2 h. If ketone level is persistently increased, consume 40 g carbohydrates and repeat in 2 h. Continue to do this until ketone levels <1.0 mmol/L. If a participant experiences >2 episodes of ketonaemia during the study, their notes will be reviewed by the PI to determine their suitability for remaining in the trial.
**Guidance for the introduction of anti‐diabetes medication** Diabetes medication will be introduced according to the following protocol: If ≥25% fasting blood glucose readings are >5 mmol/L and/or ≥25% of 1 h postprandial glucose readings are >7 mmol/L in a 7 day period: Commence metformin MR 500 mg daily Increased every 3 days by 500 mg–1 g BD (if tolerated) If after reaching optimal or maximum tolerated dose of metformin ≥25% fasting blood glucose readings remain >5 mmol/L in a 7‐day period: Commence bedtime isophane insulin 4 units and uptitrate the dose by 2 units every 3 days aiming for a fasting glucose of ≤5 mmol/L, And/or ≥25% of 1 h postprandial glucose readings are >7 mmol/L in a 7 day period: Commence prandial fast acting insulin analogue (Humalog or Novorapid) 2–4 units with the relevant meal. Uptitrate the dose by 2 units every 3 days aiming for a 1 h postprandial glucose of ≤7 mmol/L. Medication adjustment will be made in accordance with the above guidance.

**FIGURE 1:**
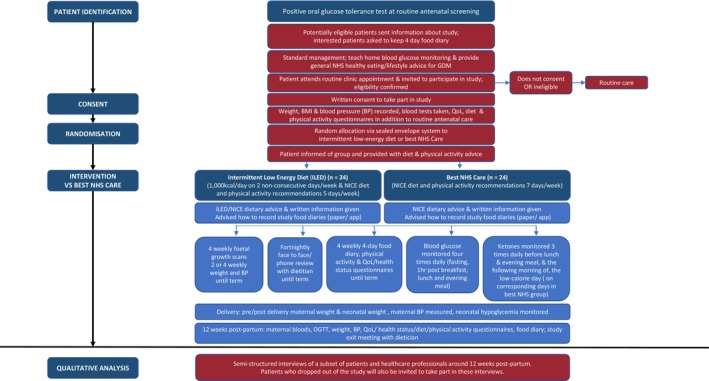
Study flow diagram.

Trial blood tests (including HbA1c, beta‐hydroxybutyrate, fasting glucose and insulin) were checked at recruitment, 34–40 weeks gestation, and 11–13 weeks postpartum. The full schedule of assessments can be found in Appendix 1 and the study flow diagram in Figure 1.

#### 2.2.3 Safety monitoring

Participants were asked to record their capillary blood glucose four times a day using Contour glucometers (Ascensia Diabetes Care UK Ltd., Berkshire), and capillary ketones 1‐h post evening meal on the ILED day and when fasting before breakfast the morning after, using NHS England approved ketone meters (A. Menarini Diagnostics, Berkshire). Participants were shown how to use the meters by their midwife as part of their routine care. The BNC group measured capillary ketones twice a week on any day of their choosing. Ketonaemia was predefined as ≥1.0 mmol/L and participants were provided with a management protocol (Table 3).

### 2.3 Measures

#### 2.3.1 Outcomes

Primary outcomes included study uptake, recruitment rate and retention, adherence to the ILED, completion of glucose and ketone self‐monitoring and safety of ILED/BNC.

Secondary outcomes included the percentage completeness of data collection for trial endpoints (Table 4).

**TABLE 4:** Outcomes.

*Primary outcomes*
Uptake rate (percentage of eligible participants who consent to take part, including the proportion of women screened who did not meet eligibility criteria, and the number of women who did not give consent to take part)
Recruitment rate (number of eligible participants who consent to take part per month)
Retention rate (number of randomised participants who complete the trial by attending the final visit and the percentage of participants who attend all eight visits)
Adherence to the dietary interventions (self‐reported adherence to the low‐energy days between randomisation and delivery)
Completion of self‐assessed glucose and ketone readings (percentage of the required readings)
*Safety outcomes*
Percentage of women following ILED/best NHS care with self‐reported hypoglycaemia (blood glucose of <3.0 mmol/mol) and/or hypoglycaemia requiring third‐party assistance
Percentage of women who develop self‐reported significant ketonaemia (≥1.0 mmol/L)
Percentage of neonatal hypoglycaemic episodes requiring intervention (blood glucose checked 2 h after birth and 2 h thereafter for 12 h according to local protocol), neonatal birth weight, gestational age at delivery, hyperbilirubinaemia/jaundice and/or admission to Special Care Baby Unit or Neonatal Intensive Care Unit, and stillbirths
Incidence and rate of other potentially diet related adverse events (i.e. headaches, lethargy, constipation or complications requiring hospital admission) between the start of the trial intervention and delivery recorded as mild, moderate and severe, as defined by Common Terminology Criteria for Adverse Events (CTCAE V.5)
Hospital admission for routine labour and delivery is not classified as an adverse event
*Secondary outcomes*
Completeness of collection of trial endpoints (percentage of completed weight measurements, 4‐day food diaries and International Physical Activity Questionnaire (IPAQ) scores)
Fidelity of delivery of interventions (measured through the number and modality of completed planned participant contacts, electronic and paper food diaries and self‐reported capillary glucose and ketone measurements)
Qualitative analysis of the acceptability and implementation of the interventions (explored amongst a subset of participants (~10 in each group) and HCPs through in‐depth interviews)
*Maternal exploratory outcomes (explored without statistical inference)*
Percentage of women requiring metformin and/or insulin
Four‐point capillary glucose profiles during the third trimester (four times daily until delivery)
Change in fasting blood test results between baseline measurements, 34–40 weeks gestation and 11–13 weeks post‐delivery (including oral glucose tolerance tests)
Mode of delivery, preeclampsia, polyhydramnios (maximum liquor volume pool depth ≥ 8 cm)
Quality of life and health status questionnaires (World Health Organisation Quality of Life (Brief Version) (WHOQoL‐BREF) and 36‐Item Short Form Survey (SF‐36) questionnaires)
*Foetal exploratory outcomes (explored without statistical inference)*
Foetal weight and gestational age at delivery

### 2.4 Patient and public involvement (PPI)

Patient and public involvement was sought throughout the planning, design and implementation of the trial through local mother and baby groups, GP clinics and word‐of‐mouth. Contributors preferred 1–1 discussions via video conferencing, and comments on study materials were returned via email. Twelve women with personal experience, or close family member experience, of GDM within the last 10 years took part in a 30–60 min discussion with a female member of the study team. Contributors represented diverse backgrounds including British, South African, Pakistani, Bangladeshi, Indian and Arab. Contributors assisted with participant materials, diet intervention design and in considering potential barriers and facilitators to recruitment. They were offered £25/h reimbursement for their time. A patient expert remained on the team throughout the study.

### 2.5 Statistical analysis

The sample size was pragmatic. Allowing for an estimated uptake of 25%, 15% attrition and aiming for complete follow‐up for 20 participants per group (40 total) based on previous dietary studies, the recruitment target was 48 participants. Assuming approximately 192 eligible participants would need to be invited to participate, this would allow uptake to be estimated with a margin of error ± 6% and retention ± 10%. The analysis is descriptive with no formal hypothesis testing. Categorical outcomes are reported as counts and percentages, with 95% confidence intervals for key feasibility outcomes, and continuous outcomes as median and interquartile range (IQR). Data were collected and managed using REDCap (Vanderbilt University, United States), an electronic data capture tool hosted at MFT.^14^

Trial progression criteria were pre‐defined (Table 5).

**TABLE 5:** Trial progression criterion.

	Feasible (green)	Feasible with modification of the protocol (amber)	Not feasible (red)
Recruitment rate	≥4 participants per month	>2 participants per month	≤2 participants per month
Uptake to the feasibility study (% of eligible participants recruited)	≥15%	10–15%	<10%
Retention to the feasibility study	>70%	50–70%	<50%
Adherence to the ILED intervention (2 days/week between 24–30 weeks and delivery)	>50% of the low energy days completed	30–50% of the low energy days completed	<30% of the low energy days completed
Number of reported adverse events	Rates in ILED <25% above those reported in BNC	Rates in ILED ≥25% above those reported in BNC	

## 3 RESULTS

### 3.1 Primary outcome measures

#### 3.1.1 Study population, uptake and retention

**FIGURE 2:**
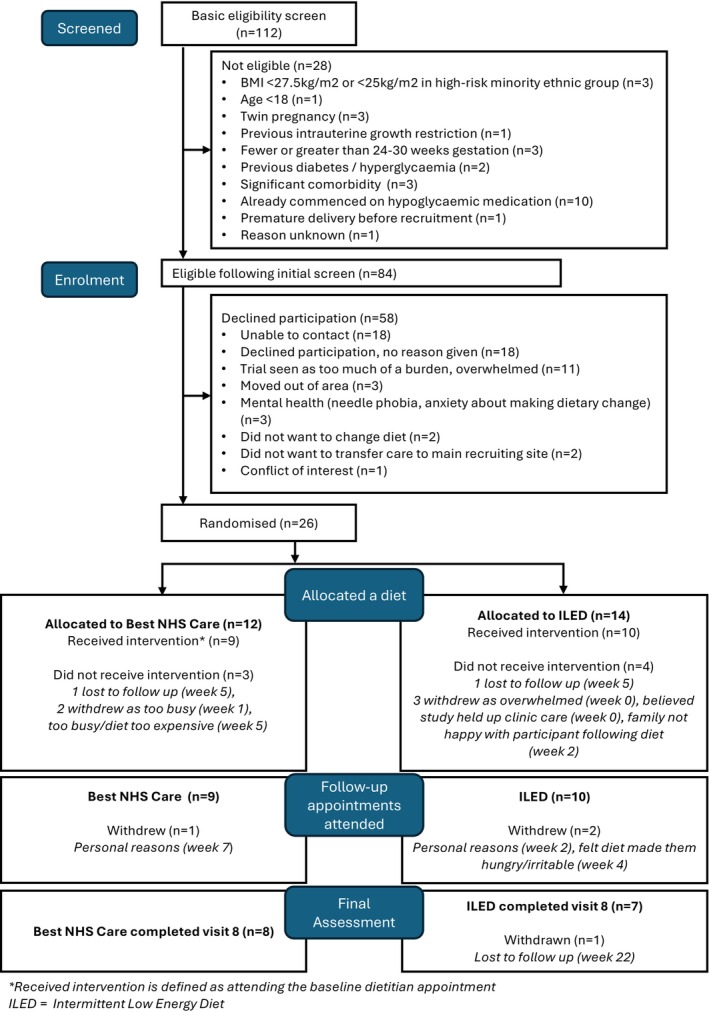
Consolidated Standards of Reporting Trials (CONSORT) flow diagram of participants recruited to the trial.

A total of 112 potential participants were screened, 84 were eligible (75%) and 26 agreed to take part (ILED *n* = 14; BNC *n* = 12) representing a 31% uptake of those eligible (95% CI: 22%–41%) and a recruitment rate of two participants per month (95% CI: 1.3–2.9) over the 13 month recruitment period. Fifteen participants (57.7%) completed the trial (95% CI: 38%–77%) and attended the final visit (50% ILED, *n* = 7; 66.7% BNC, *n* = 8). Reasons for declining participation and/or withdrawal included feeling overwhelmed, family pressures and trial procedures considered too burdensome in addition to usual care (Figure 2).

#### 3.1.2 Participant characteristics

Participant characteristics are detailed in Table 6. The median age of all participants was 34 years with 42.9% (*n* = 6) of women in ILED group and 50% (*n* = 6) in BNC group in the most deprived deciles of the index of multiple deprivation (IMD‐1‐2).^15^ No participants had previous GDM. The characteristics of our participant group broadly reflect the general population with GDM at the recruiting centre during the trial period, with comparable age, BMI and deprivation score (Table 6). The recruited cohort were more likely to be married and Asian than the overall population managed in the unit that year. Participants who completed the study were more likely to be Asian and from more deprived areas than those who withdrew (Supporting Information Appendix 1).

**TABLE 6:** Participant characteristics of the intermittent low‐energy diet (ILED), best NHS care (BNC) groups and overall GDM population at the recruiting unit during the recruitment period.

Characteristic	All study participants (*n* = 26)	ILED (*n* = 14)	BNC (*n* = 12)	Overall GDM population in recruiting clinic (*n* = 264)
Age, years (median and IQR)	34.00 [30.00, 36.00]	34.00 [31.00, 36.00]	33.00 [29.00, 35.50]	34.0 [29.0, 37.0]
Marital status *n* (%)				
Living alone: single	1 (4.0)	0 (0.0)	1 (8.3)	64 (24.2)
Living with partner	24 (96.0)	13 (100.0)	11 (91.7)	181 (68.5)
Missing data (*n*)	1	1	0	19
Children < 18 years living at home *n* (%)				
No	12 (50.0)	6 (46.2)	6 (54.5)	
Yes	12 (50.0)	7 (53.8)	5 (45.5)	
Missing data (*n*)	2	1	1	
Number children < 18 years living at home *n* (%)				
0	12 (50.0)	6 (46.2)	6 (54.5)	
1	4 (16.7)	4 (30.8)	0 (0.0)	
2+	8 (33.3)	3 (23.1)	5 (45.5)	
Level of education *n* (%)				
‘O’ levels/GCSEs	2 (9.5)	0 (0.0)	2 (18.2)	
‘A’ levels/other post‐16 qualifications at college	4 (19.0)	3 (30.0)	1 (9.1)	
Degree	11 (52.4)	5 (50.0)	6 (54.5)	
Postgraduate qualification (highest level), e.g. Masters, PhD	4 (19.0)	2 (20.0)	2 (18.2)	
Missing data (*n*)	5	4	1	
Employment status *n* (%)				
Paid work full time (>30 h/week)	17 (70.8)	9 (69.2)	8 (72.7)	
Paid work part time (<30 h/week)	4 (16.7)	1 (7.7)	3 (27.3)	
Full‐time student	1 (4.2)	1 (7.7)	0 (0.0)	
Not working: unemployed	2 (8.3)	2 (15.4)	0 (0.0)	
Missing data (*n*)	2	1	1	
In receipt of welfare benefits *n* (%)				
No	21 (91.3)	12 (92.3)	9 (90.0)	
Yes	2 (8.7)	1 (7.7)	1 (10.0)	
Missing data (*n*)	3	1	2	
Ethnicity *n* (%)				
White (British/Other)	11 (44.0)	6 (46.2)	5 (41.7)	139 (52.7)
Asian/Asian British (Indian/Pakistani/Other)	11 (44.0)	6 (46.2)	5 (41.7)	79 (30.0)
Black/Black British (Caribbean)	1 (4.0)	0 (0.0)	1 (8.3)	19 (7.1)
Mixed White/Black (African/Caribbean)	2 (8.0)	1 (7.7)	1 (8.3)	27 (10.0)
Missing data (*n*)	1	1	0	0
Smoking history *n* (%)				
Current smoker	2 (8.0)	0 (0.0)	2 (16.7)	
Ex‐smoker	4 (16.0)	4 (30.8)	0 (0.0)	
Never smoker	19 (76.0)	9 (69.2)	10 (83.3)	
Missing data (*n*)	1	1	0	
Parity *n* (%)				
0	12 (48.0)	6 (46.2)	6 (50.0)	102 (39.1)
1	4 (16.0)	4 (30.8)	0 (0.0)	94 (35.6)
2+	9 (36.0)	3 (23.1)	6 (50.0)	58 (22)
Missing data (*n*)	1	1	0	10
Index of deprivation (median and IQR)^a^	3.00 [1.00, 5.00]	3.00 [2.00, 4.00]	3.00 [1.00, 7.00]	
Deprivation centile 1–2 *n* (%) most deprived	12 (46)	6 (42.9)	6 (50.0)	100 (38)
Deprivation centile 3–4 *n* (%)	6 (23)	5 (35.7)	1 (8.3)	47 (18)
Deprivation centile 5–6 *n* (%)	2 (8)	1 (7.1)	1 (8.3)	29 (11)
Deprivation centile 7–8 *n* (%)	3 (12)	0 (0.0)	3 (25.0)	44 (17)
Deprivation centile 9–10 *n* (%) least deprived	3 (12)	2 (14.3)	1 (8.3)	44 (17)
Height (cm; median and IQR)	160.5 [156.0,164.8]	160.5 [156.0, 163.4]	160.5 [156.0, 165.5]	
Booking BMI^b^ (kg/m^2^; median and IQR)	31.8 [29.4, 35.3]	31.5 [29.6, 35.1]	32.1 [29.4, 35.6]	30.0 [25.0, 36.0]
Booking weight^b^ (kg; median and IQR)	82.2 [77.3, 101.9]	82.2 [77.3, 92.2]	82.5 [76.5, 107.0]	
Booking gestational week (median and IQR)	10.1 [9.1, 11.5]	10.1 [9.6, 11.2]	9.7 [8.4, 11.9]	

^a^ Index of deprivation calculated using Geoconvert.^15^

^b^ Booking weight and BMI taken from electronic health records.

#### 3.1.3 Self‐reported dietary adherence and completion of glucose and ketone monitoring for study completers

Completers in the ILED group (*n* = 7) reported undertaking a median (IQR) 31 (6%–45%) of the expected low‐energy days between recruitment and delivery. Neither group reported adopting another diet during the study. No participants continued with ILED after delivery.

Collectively all completers (*n* = 15) completed median (IQR) 73 (48–90)% of required glucose measurements and 18 (2–38)% of required ketone measurements.

#### 3.1.4 Safety outcomes across all trial participants in the study

Maternal and neonatal adverse events (AEs) are reported in Table 7. There were few safety concerns with the ILED and BNC interventions in terms of hypoglycaemia and ketonaemia. Five serious adverse events (SAEs) were reported amongst participants; three in ILED and two in BNC. None of the SAEs or AEs in mothers or neonates were considered related to the dietary interventions.

**TABLE 7:** Maternal and neonatal adverse events.

ILED (*n* = 8)	Best NHS care (*n* = 8)
Blurred vision	Blurred vision
Headache	Headache × 2
Polyhydramnios	Polyhydramnios
Reduced foetal growth velocity	Foetal growth restriction
Hunger and irritability	Hypertension in pregnancy × 3
Reduced foetal movements	Oedema × 2
Maternal tachycardia	Gingival pain
Myalgia	Flu like symptoms
Intermittent vaginal bleeding	Upper respiratory tract infection
Urinary tract infection	Pain in extremity
Neonatal cyst on kidney	Pre‐eclampsia (medication controlled)
Neonatal hydronephrosis	Neonatal jaundice
Postpartum episiotomy infection	
Postpartum constipation	
Postpartum endometritis	
Neonatal hypoglycaemia	
Neonatal jaundice	
Neonatal admission to Special Care Baby Unit/Neonatal Intensive Care Unit	

*Note*: Adverse events are listed as any event reported by participants in either group. Participants could report multiple adverse events at each time point. Eight participants from ILED and 8 participants from best NHS care reported any adverse events.

### 3.2 Secondary outcomes

#### 3.2.1 Completeness of study data across all trial participants

Completeness of data for maternal weight, food diaries and study questionnaires are reported in Supporting Information Appendix 2. Missing data were largely due to participants withdrawing from the study, however, maternal weight was not always recorded as many reviews were virtual or weights were missed in busy clinics. Only five of the cohort (19%) opted to use the Nutritics Libro app (Nutritics, Dublin, Ireland; https://www.nutritics.com) to record food diaries, others used paper. There was poor completion of food diaries across all timepoints.

#### 3.2.2 Fidelity of delivery of the dietary intervention

Twelve of the 14 ILED and 10 of the 12 BNC participants received initial dietary advice which required a median (IQR) of 60 (52–60) min of dietitian time for ILED and 60 (30–70) min for BNC participants. Nine ILED and seven BNC participants attended follow‐up calls lasting 31 (15–50) min for ILED and 40 (26–45) min for BNC participants. During pregnancy ILED participants had 3.6 dietitian calls (33% video/73% telephone) and BNC participants a median of five calls (55% video/45% telephone). Six ILED participants had one‐four additional emails, and five BNC had one‐two additional emails. Only one ILED and two BNC participants wanted a 12‐week postpartum dietitian review. Most contacts were via phone/video call; only two baseline diet advice sessions were face‐to‐face.

### 3.3 Exploratory outcomes

Exploratory outcomes are reported for completers in the study ILED (*n* = 7)/BNC (*n* = 8).

#### 3.3.1 Anti‐diabetes medication

More participants in the ILED group received anti‐diabetes medication. Five (71.4%) were treated with metformin and two (28.6%) received metformin and insulin. Three of the BNC group (37.5%) received no medication, two (25%) were treated with metformin, and three (37.5%) received metformin and insulin. One participant in the BNC group declined medication despite clinical advice.

#### 3.3.2 Maternal weight blood pressure and glycaemic control

Change in weight, blood pressure, four‐point capillary glucose profiles, HbA1c and insulin are reported in Table 8. Both groups were weight‐steady during the last trimester and had reduced weight 12 weeks postpartum. HbA1c increased in both groups in the last trimester of pregnancy and returned to baseline postpartum. Insulin and insulin resistance were higher in the ILED group postpartum, although the sample was small with wide ranges. There were no notable differences in median fasting and 2‐h OGTT results at baseline (~24–30 weeks gestation) and 12 weeks postpartum (Table 8).

**TABLE 8:** Change in maternal weight, blood pressure and HbA1c for trial completers from antenatal booking to final study visit, and glucose profiles, gestational week of delivery and birthweights for completers in both groups.

Outcome	Visit	All (*n* = 13)		ILED (*n* = 5)		BNC (*n* = 8)	
		Median (IQR)	Change from previous time point	Median (IQR)	Change from previous time point	Median (IQR)	Change from previous time point
Weight (kg)	Booking	82 (76.3–94.5)		84 (76.3–101.5)		82 (76.5–90.2)	
	GW 24–30	90.8 (84.1–102)	6.6 (8.4, 5.4)	93.2 (86.5–107.3)	7.5 (9.3, 5.1)	89.4 (83–99.4)	6.2 (8.3, 5.4)
	GW 30–36	89.5 (82.6–103)	0.7 (−1.7, 1)	94 (87.2–106.8)	0.7 (−0.9, 0.8)	86.9 (82–99.3)	0.2 (−1.7, 1.2)
	GW 34–40	90.8 (84.3–104.2)	0.8 (0.2, 1.8)	94.1 (86.8–107.2)	0.3 (−1.1, 0.6)	89.5 (83.8–101.3)	1.1 (0.4, 2.9)
	WPP 11–13	82.6 (79.4–95.3)	−5.1 (−8, −3.6)	87.4 (82.3–99.4)	−7.2 (−8, −5.7)	81.2 (77.2–94.2)	−5 (−7, −3.6)
Diastolic blood pressure (mmHg)	GW 24–30	71 (64–72)		71 (63–72)		68.5 (65.5–73.5)	
	GW 30–36	70 (59–75)	−2 (−7, 4)	59 (56–61)	−7 (−12, −2)	73 (67.8–75.5)	2 (−3, 4.2)
	GW 34–40	72 (65–77)	3 (−5, 11)	69 (58–72)	1 (−15, 6)	75.5 (71.8–81)	6 (0.2, 11.2)
	WPP 11–13	72 (68–85)	8 (−1, 12)	70 (68–71)	7 (−1, 12)	74 (70.5–86)	9.5 (−0.8, 13.8)
Systolic blood pressure (mmHg)	GW 24–30	121 (120–125)		121 (112–121)		122 (120.8–127)	
	GW 30–36	120 (113–131)	−1 (−4, 1)	117 (113–130)	1 (−4, 1)	120.5 (115.8–131.5)	−2.5 (−9, 1.8)
	GW 34–40	126 (121–133)	3 (−4, 9)	124 (121–126)	3 (1, 5)	129.5 (122.2–133)	0 (−6.2, 12.2)
	WPP 11–13	125 (119–136)	2 (−4, 13)	125 (119–128)	4 (−4, 7)	130 (118.5–136)	−1 (−5, 13.5)

		All (*n* = 10)		ILED (*n* = 6)		BNC (*n* = 4)	
HbA1c mmol/mol (%)	GW 24–30	36 (34–38) [5.4 (5.3–5.6)]		37 (35–38) [5.5 (5.4–5.6)]		35 (33–38) [5.4 (5.2–5.6)]	
	GW 34–38	38 (36–40) [5.6 (5.4–5.8)]	2 (−1, 5) [2.3 (−2.2, 2.6)]	37 (36–38) [5.5 (5.4–5.6)]	2 (−1, 3) [2.3 (−2.2, 2.4)]	40 (38–41) [5.8 (5.6–5.9)]	5 (2, 6) [2.6 (2.3, 2.7)]
	WPP 11–13	38 (35–40) [5.6 (5.4–5.8)]	2 (0, 3) [2.3 (0.0, 2.4)]	38 (36–40) [5.6 (5.4–5.8)]	2 (1, 3) [2.3 (2.2, 2.4)]	36 (33–40) [5.4 (5.2–5.8)]	1 (−1, 2) [2.2 (−2.2, 2.3)]

		ILED		BNC	
		*n*	Median (IQR)	*n*	Median (IQR)
Daily 4 point glucose readings	Readings ‘in range’ (3.9–7.8 mmol/L) *n* (%)	*n* = 5	89 (67.0–91.5)	*n* = 7	90 (80.0–93.0)
	Glucose readings >7.8 mmol/L *n* (%)	*n* = 5	10.5 (81.‐32.4)	*n* = 7	7.8 (7.0–19.2)
	Glucose readings <4.0 mmol/L *n* (%)	*n* = 5	0.5 (0.0–0.8)	*n* = 7	1.1 (0.0–1.5)
OGTT results	Fasting GW 24–30 (mmol/L)	*n* = 14	5.5 (5.3–5.7)	*n* = 12	5.5 (5.0–6.1)
	2 h GW 24–30 (mmol/L)	*n* = 14	7.2 (5.9–9.8)	*n* = 12	8.6 (7.9–9.6)
	Fasting 12 weeks postpartum (mmol/L)	*n* = 7	6.2 (5.7–8.1)	*n* = 8	6.0 (5.8–7.8)
	2 h 12 weeks postpartum (mmol/L)	*n* = 7	5.8 (5.2–6.0)	*n* = 8	5.1 (4.7–5.6)
Fasting glucose	12 weeks postpartum fasting glucose (mmol/L)	*n* = 7	5.8 (4.8–6.2)	*n* = 8	5.1 (3.9–7.3)
Insulin	12 weeks postpartum insulin (pmol/L)	*n* = 7	71 (57–156)	*n* = 8	58.5 (39–136)
HOMA‐IR	12 weeks postpartum HOMA‐IR	*n* = 7	3.1 (2.0–7.1)	*n* = 8	2.2 (0.9–4.9)
Gestational week of delivery		*n* = 7	39.3 (39.0–39.7)	*n* = 8	38.6 (37.3–39.8)
Birthweight (g)		*n* = 7	3245 (2940–3495)	*n* = 8	3650 (2713–4106)
Birthweight centile^a^		*n* = 7	39 (20–58)	*n* = 8	75 (17–95)
Birthweight *z* score^a^		*n* = 7	−0.28 (−0.86–0.18)	*n* = 8	0.77 (−1.08–1.6)

*Note*: The average gestational week at antenatal booking for all participants who completed each visit was 10 + 6 weeks. The average gestational week at antenatal booking was 11 + 6 in the ILED group and 9 + 6 in the BNC group.

Abbreviations: BNC, Best NHS Care; GW, gestational week; HOMA‐IR, Homeostatic Model Assessment for Insulin Resistance; ILED, intermittent low‐energy diet; IQR, interquartile range; OGTT, oral glucose tolerance test; WPP, weeks postpartum.

^a^ The birthweight centile and *z* score were calculated using The Fetal Medicine Foundation online calculator (https://fetalmedicine.org).

#### 3.3.3 Mode of delivery, gestational age at birth and birth weight

There were no normal vaginal deliveries in the ILED group; two required instrumental delivery, two were elective caesarean sections, and three were emergency caesarean sections. One delivery in the BNC group was a normal vaginal delivery, two required instrumental delivery, three were elective caesarean sections, and two were emergency caesarean sections.

#### 3.3.4 Foetal weight and neonatal measurements

Foetal measurements from antenatal scans are reported in Supporting Information Appendix 3, demonstrating comparable growth between groups. Birth data were available for eight ILED and eight BNC and is reported in Table 8. The BNC group had a higher median birth weight with a greater spread of birth weights than the ILED group.

#### 3.3.5 Questionnaire data/food diaries

Questionnaire data for quality‐of‐life and diet quality are reported in Supporting Information Appendix 4. Low completion rates limit their utility, but they are reported for completeness. Both groups have improved diet quality scores during pregnancy; however, scores returned to baseline level at 12 weeks postpartum, suggesting the improvements during pregnancy were not maintained postpartum. Dietary intake from food diaries completed at baseline and week 30–40 during pregnancy are reported for 10 participants (ILED *n* = 4; BNC *n* = 6), showing reduced carbohydrate, free sugar, energy and saturated fat, with maintained protein and monounsaturated fat intake, and maintained but low intakes of fibre.

## 4 DISCUSSION

The current ILED and study design do not fulfil our a priori eligibility criteria for progression to a definitive RCT, with low study retention and adherence to ILED. The study achieved a feasible uptake rate; however, recruitment was lower than expected due to a smaller than anticipated pool of eligible participants. These findings are consistent with wider literature demonstrating variable retention rates and dietary adherence during pregnancy.^16^, ^17^, ^18^, ^19^

Safety outcomes were reassuring with no women experiencing serious adverse events related to the ILED. This should be interpreted cautiously given the small sample size and low adherence rates; however, there were no significant adverse events in adherent participants. These findings align with previous research showing modest restricted caloric intake in pregnancy is not associated with significant adverse outcomes.^20^

Higher completion rates were seen among women who identified as Asian/Asian British (Indian/Pakistani/Other), and among women with lower deprivation scores, which is contrary to previous weight control research.^21^ A number of those completing the trial were South Asian nurses living in lower sociodemographic areas which is likely to skew the findings as preexisting healthcare knowledge may improve motivation for adherence. More women who withdrew had one or more children compared to those who completed the trial, likely reflecting competing priorities and time constraints making dietary trial participation difficult. This echoes previous research demonstrating that childcare and household responsibilities are a barrier to adherence, limiting self‐prioritisation for women who often shoulder the burden of multiple responsibilities.^17^ Correspondingly a greater number of those who withdrew were working full‐time.

Adherence to the ILED in this study was comparable to those of people with T2D asked to follow an ILED for weight loss (median 35%; IQR 21%–49%).^22^ Interestingly, adherence rates in other patient groups have been considerably higher; for example, Harvie et al. report adherence to ILED of 76% for women at risk of breast cancer with overweight/obesity and 77% during adjuvant chemotherapy for breast cancer.^23^ Such differences may reflect variable motivation to engage in health‐promoting dietary behaviours across populations with distinct medical conditions. Although pregnancy can be a time for heightened motivation for healthy behaviour change, the physical and psychological changes of pregnancy, the short time period in which to implement changes and competing priorities are likely to impact adherence in this cohort. This aligns with existing literature identifying pregnancy as a particularly challenging time for adopting dietary change.^24^

Moreover, any beneficial dietary changes made during pregnancy were not maintained postpartum and postpartum engagement with the dietitian was poor. This likely represents competing priorities and insufficient clinical support postpartum and limited awareness of ongoing cardiometabolic risk. These challenges may also reflect broader issues in postpartum care delivery including fragmented services and unclear responsibility for ongoing care. Previous studies have reported a sense of ‘abandonment’ after birth, and highlight the critical need for ongoing support for women with a history of GDM to facilitate meaningful, long‐term healthy behaviour change.^25^ Huang et al found individualised goal‐setting a successful way to motivate behaviour change in women with a history of GDM, and emphasized that a seamless transition of care from pregnancy to postpartum is essential for sustained change.^26^ Currently, the care pathway does not align with this model: women receive intensive care throughout pregnancy and are seen only once by their GP between 6–8 weeks postpartum.^27^ Although postpartum diabetes screening is recommended in primary care (HbA1c/fasting plasma glucose), uptake is often poor due to limited clinician and patient awareness.^28^, ^29^ A more integrated approach to care and flexible timing of delivery of interventions in high‐risk pregnancies may improve attrition and long‐term outcomes.

Most participants in both groups required anti‐diabetes medication. This may reflect difficulties adhering to the advised dietary regimes or higher insulin resistance due to overweight/obesity or ethnicity. These findings are consistent with the low‐quality studies reviewed by Yamamoto et al. showing no reduction in anti‐diabetes medication using low carbohydrate or energy restricted diets in GDM.^5^

Lower than expected completion rates of questionnaires and food diaries highlight the need to reduce the burden of trial procedures among women experiencing medically complex pregnancies.

Our findings suggest that implementing ILED in the third trimester in people with GDM may not be feasible. Although dietary interventions may have the potential to improve maternal glycaemic control, their introduction during pregnancy may limit adherence and efficacy.^5^, ^17^, ^19^ Recently the DiGest trial found provision of calorie‐controlled meals to participants reduced long‐acting insulin requirements in GDM, however there was no difference in the need for metformin or short‐acting insulin and no difference in gestational weight gain.^20^ Women who lost weight were more likely to have better glucose control and postpartum HbA1c and less likely to have large for gestational age babies, suggesting that weight loss in maternal obesity may be key to improving cardiometabolic parameters in GDM rather than the specific type of dietary intervention.^20^

Over half of our participants were from a minority ethnic group; this is important as sociocultural beliefs about diet and exercise in pregnancy can differ between and within cultures.^30^ Future research should consider a wider sample using a multisite approach and consider the need for tailored interventions for different cultural groups.

Strengths of the study include that it is the first to evaluate an ILED in the treatment of GDM, adding to the limited evidence base on dietary interventions during pregnancy. The cohort was diverse, including a wide range of ethnicities and deprivation centiles. The mixed‐methods design is a key strength, and the qualitative component of the study will provide meaningful insight into potential barriers and facilitators of intervention delivery and adherence.

Limitations include underrecruitment and a high withdrawal rate. Fidelity to the study protocol was impacted by incomplete data collection and missing trial data. The limited sample size also precluded meaningful comparison between subgroups based on demographic characteristics.

In conclusion this study suggests that an ILED intervention is unlikely feasible in the third trimester. Future research should explore alternative approaches to the prevention and management of GDM, including optimising preconception health, earlier screening for GDM in high‐risk populations, and the initiation of dietary and weight management strategies earlier in high‐risk pregnancies. An individualised approach targeting women at highest risk of GDM, including those from high‐risk ethnic groups, may be particularly important given the heterogeneity in GDM risk, variability in metabolic phenotypes, and diverse cultural and socioeconomic contexts, all of which may influence the effectiveness and acceptability of dietary interventions.

## AUTHOR CONTRIBUTIONS

ED and MH wrote the manuscript with input from all authors. BI and MH were PIs for the project and maintained overall oversight and responsibility. WM, JA and ED recruited participants and saw them for study visits. BI was responsible for the overall clinical care of each participant. MH, CL and AV were the study dietitians. EB was responsible for statistical analysis. AP and FH provided specialist advice for obstetrics and diabetes respectively. BM oversaw qualitative methodology and analysis. LD is a medical student who collected and analysed data.

## FUNDING INFORMATION

This trial is funded by the National Institute for Health Research (NIHR201944) and sponsored by Manchester University NHS Foundation Trust (MFT). The funders of the study had no role in the study design or writing of the report. Dr. Dapre is an NIHR‐sponsored GP academic clinical fellow.

## CONFLICT OF INTEREST STATEMENT

Michelle Harvie has coauthored three self‐help books for the public to follow intermittent diets. All author proceeds are paid directly to the charity Prevent Breast Cancer (registered charity number 1109839) to fund breast cancer research.

## Supporting information


**Appendix 1:** Supplementary Appendix.

**Appendix 2:** Supplementary Appendix.

**Appendix 3:** Supplementary Appendix.

**Appendix 4:** Supplementary Appendix.

**Appendix 5:** Supplementary Appendix.

## APPENDIX 1

**Table:**

Gestational week	Schedule of assessments				
	24–30	30–34	34–40	Delivery	Weeks postpartum 11–13
Eligibility and informed consent	X				
Randomisation	X				
Tailored dietitian review (face to face or remote)	X	X	X		X
Height	X				
Weight^a^	X	X	X	X	X
Blood pressure^a^	X	X	X	X	X
Fasting blood sample^b^	X		X		X
Questionnaires^c^	X	X	X		X
4‐day food diary	X		X		X
Foetal growth scan	X	X	X		
Review of glucose and ketone measurements	X	X	X		
Neonatal measurements^d^				X	
Oral glucose tolerance test					X
Exit interview/end of study questionnaires					X
Invitation to optional qualitative sub‐study^e^					X

^a^ Frequency of assessment will be 2–4 weekly depending on whether appointment is face‐to‐face or virtual due to COVID‐19 restrictions.

^b^ Fasting bloods: urea and electrolytes, liver function tests, bone profile, lipids, thyroid function tests, HbA1c, beta‐hydroxybutyrate, free fatty acids, full blood count, fasting glucose, insulin.

^c^ Questionnaires: World Health Organisation Quality of Life (brief version), 36‐Item Short Form Survey, International Physical Activity Questionnaire (short form), UK Diabetes and Diet Questionnaire.

^d^ Neonatal measurements include gestational age at delivery, mode of delivery, and neonatal weight.

^e^ Sub‐study involves semi‐structured interviews exploring thoughts and experiences of the trial.

**Table:**

Dietitian led intermittent low‐energy diet and best NHS care interventions			
Timeline	Dietary advice	ILED	Best NHS Care
After initial recruitment week 24–30	Standard ‘stop gap’ advice for the best NHS care diet before randomised i.e. Increased fruit/vegetable, low‐glycaemic index, reduced free sugars, regular meals, at least 70g protein/28g fibre, predominantly mono‐ and polyunsaturated fats	x	x
Between randomisation (weeks 24–30) and delivery	The best NHS care diet was personalised for each participant including guidance on maximum energy intake (24 kcal/kg body weight if BMI <30 kg/m^2^ or 18 kcal/kg body weight if BMI >or = 30 kg/m^2^) The recommended number of portions of carbohydrate, protein and fat foods to respectively provide 45%, 25% and 30% of total intake as recommended in several GDM dietary guidelines	5 days/week	7 days/week
	Food based low‐energy days (1000 kcal including 100g low‐GI carbohydrate and 70g of protein)	2 non‐ consecutive days/week	
	Provided menus, recipes and meal plans for their allocated diet	x	x
	**Physical activity advice**	x	x
	~150 min moderate intensity physical activity/week	x	x
	Walking for 30 min after mealtimes wherever possible	x	x
	**Dietetic education and support**	x	x
	One to one personalised written and verbal advice (phone/video call ~45 min)	x	x
	2 weekly reviews^a^	x	x
	Phone/video call/email 15–30 min		
	**Participant Monitoring**	x	x
	4‐day diet diaries (Libro Nutritics or paper diaries) at baseline, weeks 34–40 and 11–13 post‐partum	x	x
	Weights at 2 weekly antenatal clinic visits	x	x
	Measure capillary glucose four times each day and ketones on two days of the week	x	x
	**Behavioural basis for the dietary interventions**		
	Both dietary interventions target three behavioural factors: Risk appraisal Raise awareness of the negative consequences of GDM Response efficacy – provide evidence for the benefits of best NHS care and ILED Self‐efficacy: dietitians used motivational interviewing which aims to promote self‐efficacy in the participant	x	x
Between delivery and final oral glucose tolerance (OGTT) test (11–13 weeks post‐partum)	Advised they can follow their allocated ILED or best NHS diet after delivery if they wish and physical activity advice, but they did not receive any further dietetic support	x	x
Final exit diet review	Dietitian discusses 12 week postnatal OGTT results. Advised they can continue to follow their allocated ILED or best NHS diet and physical activity advice if they wish. Recommend referral to the NHS Diabetes Prevention Programme		

^a^ Participants in either group could have weekly reviews if dietitians felt they were required or if they were requested by participants.
