# Supplementary material for: Testing the feasibility of an intermittent low‐energy diet in women with gestational diabetes
Source: Diabet Med. 2026 Mar 20;43(7):e70258. doi: 10.1111/dme.70258 (PMC13257903; doi:10.1111/dme.70258)
Supplement: Supplementary file 1 — Appendix 1: Supplementary Appendix. [file DME-43-e70258-s004.docx]

| **Characteristic** | | **Completed** | **Withdrew/**  **lost to follow up** |
| --- | --- | --- | --- |
|  |  | **(n=15)** | **(n=11)** |
| **Age, years (median and IQR)** | | 32.0 [30.0, 35.5] | 34.0 [32.0, 35.8] |
| **Marital status n (%)** | Living alone: Single | 0 (0.0) | 1 (10.0) |
|  | Living with partner/others | 15 (100.0) | 9 (90.0) |
|  | Missing data (n) | 0 | 1 |
| **Number of children <18 years living at home n (%)** | 0 | 9 (60.0) | 3 (33.3) |
|  | 1 | 3 (20.0) | 1 (11.1) |
|  | 2+ | 3 (20.0) | 5 (55.6) |
|  | Missing data (n) | 0 | 2 |
| **Education n (%)** | 'O' Levels / GCSEs | 1 (7.7) | 1 (12.5) |
|  | 'A' Levels /other post-16 qualifications at college | 1 (7.7) | 3 (37.5) |
|  | Degree | 8 (61.5) | 3 (37.5) |
|  | Postgraduate qualification (highest level), e.g  Masters, PhD | 3 (23.1) | 1 (12.5) |
|  | Missing data (n) | 2 | 3 |
| **Employment status n (%)** | Paid work full time (more than  30 hours a week) | 10 (66.7) | 7 (77.8) |
|  | Paid work part time (less than  30 hours a week) | 3 (20.0) | 1 (11.1) |
|  | Not working: unemployed | 1 (6.7) | 1 (11.1) |
|  | Full Time Student | 1 (6.7) | 0 (0.0) |
|  | Missing data (n) | 0 | 2 |
| **In receipt of welfare benefits n (%)** | Yes | 1 (6.7) | 1 (12.5) |
|  | No | 14 (93.3) | 7 (87.5) |
|  | Missing data (n) | 0 | 3 |
| **Ethnicity n (%)** | White (British/Other) | 4 (26.7) | 7 (70.0) |
|  | Asian/Asian British (Indian/  Pakistani/Other) | 9 (60.0) | 2 (20.0) |
|  | Black/Black British (Caribbean) | 0 (0.0) | 1 (10.0) |
|  | Mixed White/Black (African/  Caribbean) | 2 (13.3) | 0 (0.0) |
|  | Missing data (n) | 0 | 1 |
| **Index of deprivation (median and IQR)* n (%)** | | 2.00 [1.00, 4.00] | 4.00 [2.25, 8.75] |
| **Previous delivery live infant n (%)** | Yes | 6 (40.0) | 7 (70.0) |
|  | No | 9 (60.0) | 3 (30.0) |
|  | Missing data (n) | 0 | 1 |

**Characteristics of participants who completed and withdrew from the study**

*Index of deprivation calculated using Geoconvert^15^; IQR=interquartile range
