# Supplementary material for: Testing the feasibility of an intermittent low‐energy diet in women with gestational diabetes
Source: Diabet Med. 2026 Mar 20;43(7):e70258. doi: 10.1111/dme.70258 (PMC13257903; doi:10.1111/dme.70258)
Supplement: Supplementary file 2 — Appendix 2: Supplementary Appendix. [file DME-43-e70258-s002.docx]

**Completeness of data for time points across the study for the 26 recruited participants**

|  | **Maternal weight** | | | | **Food diaries** | | | | **IPAQ** | | | |
| --- | --- | --- | --- | --- | --- | --- | --- | --- | --- | --- | --- | --- |
|  | Complete | Missing data | Withdrew | Not measured | Complete | Missing data | Withdrew | Not completed | Complete | Missing data | Withdrew | Not completed |
| **GW 24-30**  **(baseline)** | 26 (100) | 0 (0) | 0 (0) | 0 (0) | 17 (65.0) | 9 (35.0) | 0 | 9 (35.0) | 17 (65.4) | 9 (34.6) | 0 (0) | 9 (34.6) |
| **GW 30-34** | 10 (38.5) | 16 (61.5) | 9 (35.0) | 7 (27.0) | 10 (38.5) | 16 (61.5) | 9 (35.0) |  | 12 (46.2) | 14 (53.8) | 9 (35.0) | 5 (19.0) |
| **GW 34-40** | 10 (38.5) | 16 (61.5) | 10 (38.5) | 6 (23.0) | 5 (19.0) | 21 (81.0) | 10 (38.5) | 11 (42.0) | 10 (38.5) | 16 (61.5) | 10 (38.5) | 6 (23.0) |
| **WPP 11-13** | 15 (58.0) | 11 (42.0) | 11 (42.0) | 0 (0) | 7 (26.0) | 19 (73.0) | 11 (42.0) | 8 (31.0) | 10 (38.5) | 16 (61.5) | 11 (42.0) | 6 (23.0) |

Completeness of data for time points across study; n (%). GW (gestational week), WPP (weeks post partum), IPAQ (International Physical Activity Questionnaire).
