# Supplementary material for: Testing the feasibility of an intermittent low‐energy diet in women with gestational diabetes
Source: Diabet Med. 2026 Mar 20;43(7):e70258. doi: 10.1111/dme.70258 (PMC13257903; doi:10.1111/dme.70258)
Supplement: Supplementary file 3 — Appendix 3: Supplementary Appendix. [file DME-43-e70258-s001.docx]

**Foetal measurements for all participants and trial completers**

|  | **Gestational Week** | **All n=24** | | **ILED n=12** | | **BNC n=12** | |
| --- | --- | --- | --- | --- | --- | --- | --- |
|  |  | **Median (IQR)** | **Change from previous**  **time point** | **Median (IQR)** | **Change from previous**  **time point** | **Median (IQR)** | **Change from previous**  **time point** |
| **Abdominal**  **Circumference**  **(mm)** | GW24-30 | 262.9 (241.8-267.4) | - | 258.6 (237-268.8) | - | 263.8 (247.9-267.4) | **-** |
|  | GW30-34 | 287.9 (278.9-309.8) | 41.2 (27-47.6) | 290.3 (283.1-309.8) | 44.2 (35.3-57.5) | 283.3 (272.1-293) | 31.4 (21.7-44.4) |
|  | GW34-38 | 321.4 (312.6-340) | 69.4 (57.5-84.6) | 316.6 (312.6-334) | 72.5 (58.2-84.8) | 326 (315.1-351.5) | 68.7 (48.4-84.6) |
| **Estimated**  **Foetal Weight Centile*** | GW24-30 | 0.7 (0.4-0.8) | - | 0.5 (0.4-0.7) | - | 0.7 (0.2-0.8) | - |
|  | GW30-34 | 0.6 (0.4-0.8) | 0 (-0.1-0.1) | 0.6 (0.4-0.8) | 0 (-0.1-0.1) | 0.5 (0.3-0.8) | 0 (-0.1-0) |
|  | GW34-38 | 0.4 (0.3-0.7) | 0 (-0.1-0.1) | 0.4 (0.3-0.6) | 0.1 (-0.1-0.2) | 0.7 (0.2-0.8) | -0.1 (-0.1-0) |
| **Estimated**  **Foetal**  **Weight (g)** | GW24-30 | 1506 (1309.2-1614.5) | - | 1408.5 (1241-1615.5) | - | 1519.5 (1357-1614.5) | - |
|  | GW30-34 | 2082 (1900-2201) | 687 (567-813) | 2129 (2033.5-2293) | 748 (698-945) | 1988 (1656.2-2181) | 572.5 (447.5-684) |
|  | GW34-38 | 2773 (2551-3115) | 1424.5 (1194.5-1765) | 2758 (2551-3030) | 1407.5 (1221-1621.5) | 3050.5 (2630.8-3415) | 1424.5 (1012.8-1765) |

IQR=interquartile range

*Based on Hadlock’s formula. (Hadlock FP, Harrist RB, Sharman RS, Deter RL, Park SK. Estimation of fetal weight with the use of head, body, and femur measurements--a prospective study. Am J Obstet Gynecol. 1985 Feb 1;151(3):333-7. doi: 10.1016/0002-9378(85)90298-4. PMID: 3881966).
