# Supplementary material for: Testing the feasibility of an intermittent low‐energy diet in women with gestational diabetes
Source: Diabet Med. 2026 Mar 20;43(7):e70258. doi: 10.1111/dme.70258 (PMC13257903; doi:10.1111/dme.70258)
Supplement: Supplementary file 4 — Appendix 4: Supplementary Appendix. [file DME-43-e70258-s003.docx]

1. **World Health Organisation Quality of Life Brief Version (WHOQOL-BREF) scores for available data for participants**

| **All Participants (WHOQOL-BREF)** | | | | | | | |
| --- | --- | --- | --- | --- | --- | --- | --- |
| **Domains** | **Gestational Week** | **All** | | **ILED** | | **BNC** | |
|  |  | **n** | **Median Score (IQR)** | **n** | **Median Score (IQR)** | **n** | **Median Score (IQR)** |
| **Environmental**  **health** | GW24-30 | n=17 | 77.1 (68.6-88.6) | n=10 | 75.7 (69.3-87.1) | n=7 | 77.1 (71.4-84.3) |
|  | GW30-34 | n=14 | 77.1 (66.4-80) | n=7 | 74.3 (65.7-80) | n=7 | 80 (72.9-80) |
|  | GW34-40 | n=10 | 80 (75-82.1) | n=3 | 77.1 (75.7-82.9) | n=7 | 80 (72.9-81.4) |
|  | WPP11-13 | n=11 | 74.3 (72.9-81.4) | n=5 | 74.3 (71.4-74.3) | n=6 | 81.4 (75.7-85) |
| **Physical**  **health** | GW24-30 | n=17 | 68.6 (60-82.9) | n=10 | 70 (57.9-86.4) | n=7 | 68.6 (62.9-78.6) |
|  | GW30-34 | n=14 | 71.4 (60.7-80) | n=7 | 62.9 (57.1-75.7) | n=7 | 71.4 (70-80) |
|  | GW34-40 | n=10 | 72.1 (57.9-78.6) | n=3 | 57.1 (57.1-71.4) | n=7 | 74.3 (65-77.1) |
|  | WPP11-13 | n=11 | 85.7 (81.4-85.7) | n=5 | 85.7 (82.9-85.7) | n=6 | 84.3 (80.7-85.7) |
| **Psychological**  **health** | GW24-30 | n=17 | 73.3 (63.3-76.7) | n=10 | 71.7 (70-84.2) | n=7 | 73.3 (63.3-75) |
|  | GW30-34 | n=14 | 70 (67.5-75.8) | n=7 | 70 (65-70) | n=7 | 73.3 (70-80) |
|  | GW34-40 | n=10 | 76.7 (67.5-76.7) | n=3 | 70 (66.7-78.3) | n=7 | 76.7 (71.7-76.7) |
|  | WPP11-13 | n=11 | 80 (71.7-85) | n=5 | 73.3 (60-80) | n=6 | 85 (80.8-86.7) |
| **Social**  **relationships** | GW24-30 | n=17 | 80 (73.3-86.7) | n=10 | 80 (73.3-86.7) | n=7 | 80 (76.7-86.7) |
|  | GW30-34 | n=14 | 80 (75-91.7) | n=7 | 80 (70-86.7) | n=7 | 80 (80-90) |
|  | GW34-40 | n=10 | 80 (80-85) | n=3 | 80 (76.7-86.7) | n=7 | 80 (80-83.3) |
|  | WPP11-13 | n=11 | 80 (80-83.3) | n=5 | 80 (80-80) | n=6 | 80 (80-85) |
| **Total score** | GW24-30 | n=17 | 75.4 (68.5-81.5) | n=10 | 73.5 (70.8-84.8) | n=7 | 75.4 (68.1-79.2) |
|  | GW30-34 | n=14 | 74.2 (71-80.5) | n=7 | 72.3 (68.1-74.2) | n=7 | 79.2 (72.7-82) |
|  | GW34-40 | n=10 | 76.2 (70.2-80.8) | n=3 | 70.8 (70-78.5) | n=7 | 76.2 (73.1-80.8) |
|  | WPP11-13 | n=11 | 79.2 (77.2-83.5) | n=5 | 78.5 (73.8-79.2) | n=6 | 82.3 (79.2-86.5) |

GW=gestational week; WPP=weeks post-partum; IQR=interquartile range

Reference: World Health Organisation. WHOQOL: Measuring Quality of Life, Available: <https://www.who.int/tools/whoqol>

1. **Short Form 36 (SF-36) scores for available data for participants from baseline to final study visit**

| **All Participants (SF-36)** | | | | | | | | | | |
| --- | --- | --- | --- | --- | --- | --- | --- | --- | --- | --- |
| **Domains** | **Visit** | **All** | | | **ILED** | | | **BNC** | | |
|  |  | **n** | **Median (IQR)** | **Change** | **n** | **Median (IQR)** | **Change** | **n** | **Median (IQR)** | **Change** |
| **Emotional wellbeing** | GW24-30 | n=17 | 68 (52-72) | - | n=9 | 64 (52-68) | - | n=8 | 76 (58-81) | - |
|  | GW30-34 | n=14 | 66 (41-76) | 0 (-8-4) | n=7 | 48 (38-72) | 4 (0-4) | n=7 | 72 (54-78) | 0 (-12-3) |
|  | GW34-40 | n=10 | 62 (56-76) | 4 (-8-4) | n=3 | 56 (48-66) | 4 (-12-8) | n=7 | 64 (58-76) | 0 (-7-4) |
|  | WPP11-13 | n=10 | 74 (56-80) | 4 (0-20) | n=4 | 62 (45-74) | 20 (10-28) | n=6 | 78 (70-80) | 2 (-9-4) |
| **Energy and Fatigue** | GW24-30 | n=17 | 30 (25-45) | - | n=9 | 25 (25-35) | - | n=8 | 35 (13.8-46.2) | - |
|  | GW30-34 | n=14 | 35 (20-63.8) | 5 (-2.5-15) | n=7 | 35 (25-50) | 0 (0-10) | n=7 | 45 (17.5-65) | 5 (-2.5-16.2) |
|  | GW34-40 | n=10 | 32.5 (21.2-51.2) | 0 (-11.7-5) | n=3 | 20 (20-47.5) | 0 (-2.5-2.5) | n=7 | 35 (27.5-47.5) | -5.8 (-17.9-18.8) |
|  | WPP11-13 | n=10 | 70 (28.8-75) | 10 (0-50) | n=4 | 50 (23.8-75) | 5 (2.5-27.5) | n=6 | 70 (47.5-77.5) | 15 (2.5-50) |
| **General Health Perception** | GW24-30 | n=17 | 60 (45-70) | - | n=9 | 60 (45-65) | - | n=8 | 60 (48.8-73.8) | - |
|  | GW30-34 | n=14 | 65 (50-73.8) | 0 (-5-5) | n=7 | 50 (37.5-65) | -5 (-5--5) | n=7 | 70 (67.5-80) | 2.5 (0-5) |
|  | GW34-40 | n=10 | 65 (57.5-77.5) | -5 (-5-5) | n=3 | 65 (52.5-72.5) | -5 (-7.5-0) | n=7 | 65 (60-75) | -2.5 (-5-3.8) |
|  | WPP11-13 | n=10 | 72.5 (61.2-83.8) | 15 (0-20) | n=4 | 60 (55-66.2) | 15 (-5-20) | n=6 | 82.5 (76.2-88.8) | 12.5 (1.2-20) |
| **Limited by Emotional Problems** | GW24-30 | n=17 | 83.3 (66.7-100) | - | n=9 | 91.7 (66.7-100) | - | n=8 | 83.3 (72.9-100) | - |
|  | GW30-34 | n=14 | 87.5 (75-100) | 8.3 (0-25) | n=7 | 100 (79.2-100) | 25 (8.3-33.3) | n=7 | 83.3 (62.5-95.8) | 4.2 (-18.8-14.6) |
|  | GW34-40 | n=10 | 62.5 (35.4-95.8) | -25 (-33.3-0) | n=3 | 25 (25-62.5) | -33.3 (-41.7--16.7) | n=7 | 75 (45.8-91.7) | -12.5 (-25-0) |
|  | WPP11-13 | n=10 | 95.8 (83.3-100) | 25 (0-33.3) | n=4 | 83.3 (75-93.8) | 33.3 (16.7-33.3) | n=6 | 100 (87.5-100) | 12.5 (0-31.2) |
| **Limited by Physical Problems** | GW24-30 | n=17 | 62.5 (37.5-81.2) | - | n=9 | 37.5 (31.2-62.5) | - | n=8 | 78.1 (60.9-82.8) | - |
|  | GW30-34 | n=14 | 50 (29.7-75) | 0 (-6.2-12.5) | n=7 | 25 (25-56.2) | 0 (0-12.5) | n=7 | 75 (50-96.9) | 0 (-28.1-9.4) |
|  | GW34-40 | n=10 | 56.2 (37.5-73.4) | -6.2 (-18.8-0) | n=3 | 25 (25-46.9) | 0 (-6.2-3.1) | n=7 | 68.8 (40.6-78.1) | -12.5 (-32.8--1.6) |
|  | WPP11-13 | n=10 | 78.1 (75-98.4) | 12.5 (-6.2-25) | n=4 | 87.5 (70.3-95.3) | 37.5 (18.8-46.9) | n=6 | 75 (75-93.8) | 3.1 (-6.2-12.5) |
| **Bodily Pain** | GW24-30 | n=17 | 45 (32.5-77.5) | - | n=9 | 37.5 (32.5-77.5) | - | n=8 | 45 (33.8-80.6) | - |
|  | GW30-34 | n=14 | 37.5 (32.5-63.1) | 0 (-8.8-6.2) | n=7 | 32.5 (27.5-61.2) | 0 (-10-0) | n=7 | 37.5 (36.2-61.2) | 6.2 (-5.6-12.5) |
|  | GW34-40 | n=10 | 45 (37.5-72.5) | 0 (0-12.5) | n=3 | 45 (38.8-61.2) | 0 (-11.2-6.2) | n=7 | 45 (37.5-67.5) | 0 (0-9.4) |
|  | WPP11-13 | n=10 | 90 (50-100) | 27.5 (-8.1-56.9) | n=4 | 75 (46.9-100) | 0 (-3.8-33.8) | n=6 | 90 (77.5-100) | 55 (-10-55) |
| **Physical Functioning** | GW24-30 | n=17 | 60 (30-75) | - | n=9 | 60 (25-70) | - | n=8 | 62.5 (50-78.5) | - |
|  | GW30-34 | n=14 | 45 (35-65) | 0 (-10-7.5) | n=7 | 35 (17.5-45) | 0 (-5-5) | n=7 | 65 (45-77.5) | 0 (-12.5-8.8) |
|  | GW34-40 | n=10 | 57.5 (32.5-77.5) | 5 (-25-15) | n=3 | 40 (35-55) | 15 (-5-30) | n=7 | 60 (42.5-80.6) | -10 (-31.7-9.7) |
|  | WPP11-13 | n=10 | 95 (72.5-98.8) | 25 (5-45) | n=4 | 82.5 (57.5-96.2) | 35 (22.5-52.5) | n=6 | 95 (83.8-98.8) | 15 (1.2-40) |
| **Social Functioning** | GW24-30 | n=17 | 50 (37.5-75) | - | n=9 | 37.5 (37.5-62.5) | - | n=8 | 56.2 (37.5-78.1) | - |
|  | GW30-34 | n=14 | 56.2 (40.6-71.9) | 12.5 (-6.2-18.8) | n=7 | 50 (37.5-62.5) | 12.5 (0-25) | n=7 | 62.5 (50-87.5) | 6.2 (-9.4-12.5) |
|  | GW34-40 | n=10 | 68.8 (50-93.8) | 0 (-25-12.5) | n=3 | 37.5 (31.2-43.8) | -25 (-25--12.5) | n=7 | 75 (68.8-100) | 6.2 (0-21.9) |
|  | WPP11-13 | n=10 | 100 (53.1-100) | 12.5 (0-25) | n=4 | 81.2 (50-100) | 25 (-12.5-37.5) | n=6 | 100 (62.5-100) | 12.5 (3.1-21.9) |
|  |  |  |  |  |  |  |  |  |  |  |
| **Physical Component Summary Score** | GW24-30 | n=17 | 61.9 (42.2-74.7) | - | n=9 | 45.6 (35-74.7) | - | n=8 | 67.3 (57-72.9) | - |
|  | GW30-34 | n=14 | 56.4 (36.4-67.5) | 0 (-9.1-4.7) | n=7 | 34.4 (28.8-57.2) | 1.2 (-8.1-3.1) | n=7 | 65.3 (56.4-71.6) | -0.3 (-7.7-7.5) |
|  | GW34-40 | n=10 | 57.5 (45.6-73.6) | -1.2 (-12.8-10) | n=3 | 45 (41.6-55.8) | 5 (-3.9-7.5) | n=7 | 63.4 (49.5-77) | -6.4 (-13.7-7.4) |
|  | WPP11-13 | n=10 | 80.6 (68.8-90.9) | 12.2 (4.4-33.1) | n=4 | 70.6 (51.2-90.1) | 11.9 (8.1-33.3) | n=6 | 80.6 (75.2-92.2) | 16.1 (2.1-29.8) |
| **Mental Component Summary score** | GW24-30 | n=17 | 56.6 (50.1-68.1) | - | n=9 | 52.8 (50.1-58.6) | - | n=8 | 61.5 (50.2-69.3) | - |
|  | GW30-34 | n=14 | 58.6 (46.1-73.8) | 5.4 (-0.5-10.3) | n=7 | 52.1 (47.9-69.2) | 7.2 (-0.4-12.5) | n=7 | 59.1 (50.5-77.6) | 4.7 (-9.5-7.4) |
|  | GW34-40 | n=10 | 60.9 (46-72.8) | -2 (-12.8-1.2) | n=3 | 31.5 (31.1-53.4) | -12.8 (-16.2--8.4) | n=7 | 61 (54-72.1) | 0.7 (-1.4-7.8) |
|  | WPP11-13 | n=10 | 80.3 (60.2-87.3) | 7.5 (1.2-26.9) | n=4 | 66 (44.3-86.7) | 7.5 (4.2-24.9) | n=6 | 80.8 (72.5-89.4) | 15.5 (2.5-26.2) |

GW=gestational week; WPP=weeks post-partum; IQR=interquartile range

Higher scores indicate better health.

Reference: Nair  R, Kachan  P. Outcome tools for diabetes-specific quality of life: Study performed in a private family practice clinic. Can Fam Physician Med Fam Can 2017:e310–5.

1. **UK Diabetes and Diet Questionnaire (UKDDQ) results for available data for participants from baseline to final study visit**

| **Gestational Week** | **All** | | | **ILED** | | | **BNC** | | |
| --- | --- | --- | --- | --- | --- | --- | --- | --- | --- |
|  | **n** | **Median score (IQR)** | **Median change (IQR)** | **n** | **Median score (IQR)** | **Median change (IQR)** | **n** | **Median score (IQR)** | **Median change (IQR)** |
| GW24-30 | n=18 | 3.8 (3.5-4) | - | n=10 | 3.8 (3.3-4) | - | n=8 | 3.8 (3.7-4) | - |
| GW30-34 | n=14 | 4.5 (4-4.7) | 0.8 (0.4-1.1) | n=7 | 4.2 (3.8-4.6) | 0.8 (0.5-1.1) | n=7 | 4.5 (4.4-4.8) | 0.6 (0.3-1.1) |
| GW34-40 | n=10 | 4.4 (4.3-4.6) | 0.6 (0.4-0.8) | n=3 | 4.5 (4.4-4.6) | 0.6 (0.5-1) | n=7 | 4.3 (4.2-4.5) | 0.5 (0.4-0.7) |
| WPP11-13 | n=11 | 3.9 (3.6-4.1) | 0.2 (0.1-0.5) | n=5 | 3.6 (3-3.7) | 0.2 (0-0.4) | n=6 | 4.1 (3.9-4.2) | 0.2 (0.2-0.5) |

GW=gestational week; WPP=weeks post-partum; IQR=interquartile range

Higher scores denote better diet quality.

Reference: England CY, Thompson JL, Jago R, et al. Development of a brief, reliable and valid diet assessment tool for impaired glucose tolerance and diabetes: the UK Diabetes and Diet Questionnaire. Public Health Nutr 2017;20:191–9. doi:10.1017/S1368980016002275

1. **International Physical Activity Questionnaire (IPAQ) short form scores for available data for participants from baseline to final study visit**

| **All Participants (IPAQ)** | | | | | | | | | | |
| --- | --- | --- | --- | --- | --- | --- | --- | --- | --- | --- |
|  | **Gestational Week** | **All** | | | **ILED** | | | **BNC** | | |
|  |  | **n** | **Median Score (IQR)** | **Change** | **n** | **Median Score (IQR)** | **Change** | **n** | **Median Score (IQR)** | **Change** |
| **Moderate activity undertaken**  **(days per week)** | GW24-30 | n=17 | 1 (0-3) | - | n=10 | 0 (0-2.2) | - | n=7 | 2 (0.5-2.8) | - |
|  | GW30-34 | n=12 | 1 (0-2.8) | -1 (-2-0) | n=6 | 2 (1-2.5) | -1 (-2.5--0.5) | n=6 | 0 (0-2) | -1 (-1.5--0.5) |
|  | GW34-40 | n=10 | 0.5 (0-2.5) | 0 (-2-2) | n=3 | 3 (3-3) | 3 (3-3) | n=7 | 0 (0-1) | -1 (-2.2-0.5) |
|  | WPP11-13 | n=10 | 6 (5-7) | 3.5 (1.8-4.2) | n=4 | - | - | n=6 | 6 (5-7) | 3.5 (1.8-4.2) |
| **Walking**  **(hours per week)** | GW24-30 | n=17 | 3.1 (1.5-10.5) | - | n=10 | 2.1 (0.5-7) | - | n=7 | 5 (2.4-12.2) | - |
|  | GW30-34 | n=12 | 1 (0.3-4.5) | -0.2 (-0.9-0.3) | n=6 | 0.2 (0-0.8) | 0.3 (0-0.8) | n=6 | 3.6 (1.5-6.2) | -0.6 (-1--0.4) |
|  | GW34-40 | n=10 | 2.8 (1.1-4.5) | -0.2 (-5.5-0.4) | n=3 | 1.5 (0.8-3.1) | 0 (-2.8-2.3) | n=7 | 3.5 (1.5-5) | -0.4 (-5.4-0.2) |
|  | WPP11-13 | n=10 | 2.5 (1.5-6.5) | -1.6 (-4.9-4.8) | n=4 | 3.5 (1.9-5.5) | 4.8 (-0.1-5.9) | n=6 | 2.5 (0.5-6) | -1.8 (-5.8--1.2) |
| **Sitting**  **(hours per day)** | GW24-30 | n=17 | 4 (2.3-6) | - | n=10 | 4 (0.8-5.8) | - | n=7 | 5 (2.6-6) | - |
|  | GW30-34 | n=12 | 4.5 (2.8-6.2) | 0 (-1.5-0) | n=6 | 6 (4.2-7.8) | 0 (0-3) | n=6 | 3 (2.2-4.5) | 0 (-2-0) |
|  | GW34-40 | n=10 | 4 (1.5-4) | 0 (-1.3-0) | n=3 | 4 (2.5-6) | 1 (0.5-2.5) | n=7 | 4 (2-4) | -1.1 (-1.8--0.2) |
|  | WPP11-13 | n=10 | 3 (2.2-5) | 0 (-0.3-2) | n=4 | 3 (2.8-4.2) | 2 (2-2.5) | n=6 | 4 (2.2-5) | -0.1 (-2.3-0) |

GW=gestational week; WPP=weeks post-partum; IQR=interquartile range

Reference: Ekelund U, Sepp H, Brage S, et al. Criterion-related validity of the last 7-day, short form of the International Physical Activity Questionnaire in Swedish adults. Public Health Nutr 2006;9:258–65. doi:10.1079/phn2005840

**Dietary intake at baseline and during the dietary interventions**

**(4-day food diaries; 10 participants: 4 ILED, 6 BNC)**

|  | **Baseline**  GW 24-30 | **During pregnancy**  GW 30–40 |
| --- | --- | --- |
| **Energy (Kj)** | 5695 (3781-7221) | 4713 (3897-6027) |
| **Carbohydrate (g)** | 138 (92-188) | 94 (72-110) |
| **Free sugar (g)** | 10 (5 – 29) | 6 (2-9) |
| **Dietary fibre (g)*** | 9 (7-15) | 9 (11-13) |
| **Protein (g)** | 73 (52-83) | 68 (59-108) |
| **Total fat (g)** | 52 (36 – 86) | 47 (36-75) |
| **Saturated fat (g)** | 18 (11 – 36) | 13 (12 -22) |
| **Monounsaturated fat (g)** | 18 (9-22) | 15 (7 -24) |
| **Polyunsaturated fat (g)** | 7 (4-12) | 7 (2-14) |

Median (interquartile range); GW=gestational week
* Englyst method
